# Cpx‐mediated amino acid sensing diversifies gastrointestinal colonization of *Klebsiella pneumoniae*

**DOI:** 10.1002/mlf2.70005

**Published:** 2025-04-23

**Authors:** Danyang Li, Qiucheng Shi, Liuqing He, Jianhua Luo, Huajie Zhu, Xiaoting Hua, Yunsong Yu, Yan Jiang, Liang Tao

**Affiliations:** ^1^ College of Life Sciences Zhejiang University Hangzhou China; ^2^ Key Laboratory of Multi‐omics in Infection and Immunity of Zhejiang Province, Center for Infectious Disease Research, School of Medicine Westlake University Hangzhou China; ^3^ School of Life Sciences Westlake University Hangzhou China; ^4^ Westlake Laboratory of Life Sciences and Biomedicine Hangzhou China; ^5^ Department of Infectious Diseases, Sir Run Run Shaw Hospital Zhejiang University School of Medicine Hangzhou China

**Keywords:** amino acid sensing, CpxRA, intestinal colonization, *Klebsiella pneumoniae*, type 3 fimbriae

## Abstract

*Klebsiella pneumoniae* is a Gram‐negative opportunistic pathogenic bacterium that occasionally inhabits the human gastrointestinal tracts. Gut‐colonized *K. pneumoniae* may then metastasize to other organs and tissues, thus causing severe infections. In this study, we identified three *cpxA* mutations in *K. pneumoniae* that experimentally evolved to show reduced adhesive ability. CpxA is a sensor histidine kinase that rendered reduced surface adhesion and gut colonization ability in *K. pneumoniae*. Interestingly, one experimentally gained CpxA mutant (L168del) also commonly occurs in nature. *K. pneumoniae* containing CpxA variants showed different colonization potentials through altered type 3 fimbriae expression. Lastly, we demonstrated that CpxA contributes to amino acid sensing, thus regulating the colonization of *K. pneumoniae* both on solid surfaces and in mouse intestines. The polymorphism of CpxA may help to broaden the environmental adaptation of the bacterium. These findings together reveal a Cpx‐mediated regulation to diversify intestinal colonization in *K. pneumoniae*.

## Impact statement

Gastrointestinal colonization is an important risk factor for severe organ or systemic infection of *Klebsiella pneumoniae*. Here, we show that the polymorphism in the sensor histidine kinase CpxA of *K. pneumoniae* leads to varied surface adhesion and gut colonization by regulating the expression of type 3 fimbriae. Furthermore, we demonstrate the role of CpxA in mediating amino acid sensing, particularly of serine and aspartic acid, which are abundant in the gut. This study elucidates the contribution of CpxA‐mediated adaptability to intestinal colonization of *K. pneumoniae*, offering new insights into bacterial pathogenicity and potentially guiding the development of new therapies.

## INTRODUCTION

*Klebsiella pneumoniae* is a Gram‐negative opportunistic pathogen belonging to the *Enterobacteriaceae* family^1^. The bacterium can infect various parts of the body, including the respiratory tract, lung, urinary tract, liver, spleen, bloodstream, soft tissue, and wound site^2^, ^3^. *K. pneumoniae* is one of the leading causes of community‐ and nosocomial‐acquired infections, which is especially malicious to vulnerable populations such as the elderly, neonates, and immunocompromised individuals^4^, ^5^.

*K. pneumoniae* is frequently detected on human body surfaces, including the skin, the nasopharynx, the respiratory tract, and the gastrointestinal tract^3^. Among these sites, gastrointestinal colonization is thought to be an important reservoir as well as a non‐negligible risk factor for transmissions and severe organ infections^6^, ^7^. Epidemiological studies have demonstrated that most nosocomial‐acquired *K. pneumoniae* infections are caused by the transmission of bacteria originally colonized in the host gastrointestinal tract. Particularly, gastrointestinal carriage of *K. pneumoniae* is strongly associated with subsequent bloodstream infection and pyogenic liver abscess in hospitals, which is now a major health problem worldwide^8^, ^9^, ^10^, ^11^.

Multiple virulence factors, including capsules, lipopolysaccharides, siderophores, and fimbriae, have been proposed to involve the mucosal colonization of *K. pneumoniae* ^12^, ^13^, ^14^, ^15^. However, how *K. pneumoniae* recognizes the environment in the lumen of the host gastrointestinal tract and adapts to colonize remains largely unclear. In this study, we managed to explore bacterial factors that contribute to the gastrointestinal sensing and colonization of *K. pneumoniae*. To achieve this, we first evaluated the surface attachment and mouse intestinal colonization of several clinically isolated *K. pneumoniae* strains. Since no obvious relationship was found between bacterial attachment and phylogeny, we then used ZJ0H289, a strain showing steady colonization in the mouse gut, to perform the experimental evolution. We found that a two‐component system sensory kinase gene *cpxA* is closely related to the attachment and intestinal colonization of *K. pneumoniae*, as CpxA variants show different signaling activities and modulate the type 3 fimbriae expression. Finally, we demonstrated that CpxA responds to amino acids such as serine and aspartic acid, which are abundant in digested food, and promotes the intestinal colonization of *K. pneumoniae*.

## RESULTS

### Assessment of surface adhesion and gut colonization of clinical *K. pneumoniae*

**Figure 1:**
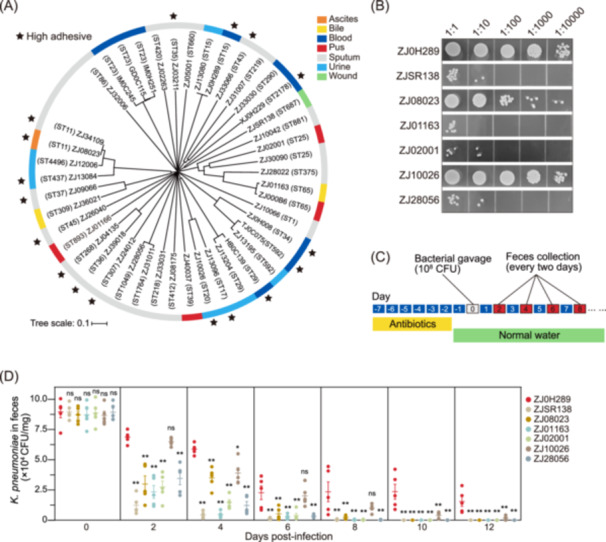
Clinical *Klebsiella pneumoniae* strains show varied surface adhesion and intestinal colonization. (A) The neighbor‐joining tree was built for 44 representative *K. pneumoniae* strains based on seven housekeeping genes (*gapA*, *infB*, *mdh*, *pgi*, *phoE*, *rpoB*, and *tonB*). The high‐adhesive strains are marked by asterisks. The isolation sites of each strain are marked with different colors. Tree scale = 0.1. (B) Serial‐diluted spotting showing the surface binding of seven clinical *K. pneumoniae* isolates. The dilution factors were 10, 100, 1000, and 10,000, respectively. (C) Schematic diagram of a mouse gavage infection model with clinical *K. pneumoniae* strains. (D) Bacterial counts of different clinical *K. pneumoniae* strains enumerated from feces. The values represent mean ± SEM, *n* = 5 mice per group, two‐tailed Mann–Whitney test. *p < 0.05; **p < 0.01; ns, not significant.

To explore factors involving gut colonization, we first evaluated the ability of surface attachment, the initial step for colonization, of clinical *K. pneumoniae* strains. Forty‐four representative *K. pneumoniae* strains, which belong to different genome multi‐locus sequence types (MLSTs)^16^ and were clinically isolated from varied infection sites, were applied to polystyrene plates to assess their surface adhesion ability. Eighteen strains showed robust adherent ability, while 26 strains showed weak binding to the plates (Figure S1). However, no overt relationship was observed between adhesion and bacterial phylogeny (Figure 1A).

We then selected three high‐adhesive and four low‐adhesive strains and tested their intestinal colonization abilities in mice^17^ (Figure 1B). Each mouse pretreated with antibiotics was inoculated with ~10^8^ colony‐forming units (CFU) of *K. pneumoniae* via oral gavage, and their feces were collected, ground, and plated onto the selective media (Figure 1C). Colony numbers were counted to estimate the amount of *K. pneumoniae* in the gastrointestinal tracts of these mice. Four low‐adhesive *K. pneumoniae* strains were largely eliminated from the gut in 4 days, while three high‐adhesive strains remained detectable in mouse feces 6 days post‐gavage (Figure 1D), confirming the strong correlation between surface adhesion and gut colonization of *K. pneumoniae*. Particularly, we found that a strain named ZJ0H289 showed relatively steady gastrointestinal colonization in mice for at least 12 days postinoculation (Figure 1D). Consistently, the *ex vivo* colonization assay in ligated intestines also showed that ZJ0H289, but not a low‐adhesive strain ZJSR138, robustly bound to the mouse intestinal epithelium (Figure S2).

### Experimental evolution reveals candidate genes for *K. pneumoniae* colonization

**Figure 2:**
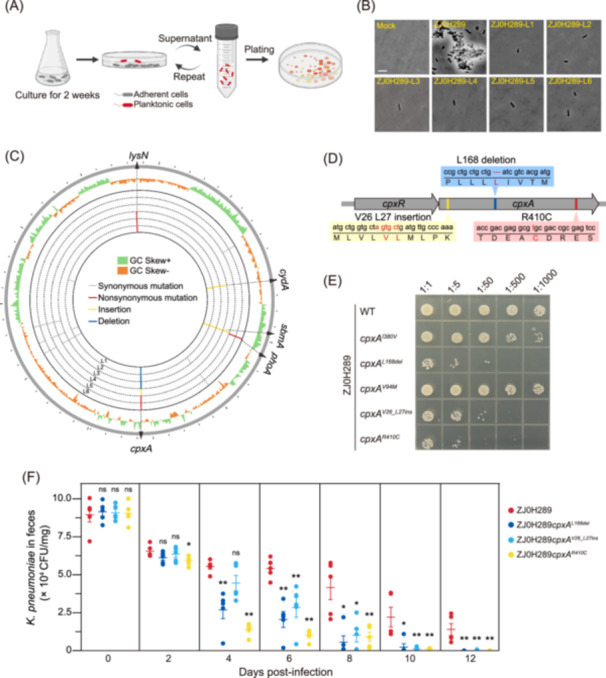
Experimental evolution reveals genes involving in *K. pneumoniae* colonization. (A) Schematic diagram of the experimental evolution for obtaining *K. pneumoniae* mutants with impaired adhesion ability (created in BioRender. Tao, L. (2025) https://BioRender.com/o34y527). (B) The representative microscopy images showing the surface binding of ZJ0H289 and its derivates on the culture plates. The scale bar represents 10 μm. (C) Whole‐genome sequence alignment of ZJ0H289 and its low‐adhesive mutants. The genome of ZJ0H289 is represented by a gray circle. The GC offset of the corresponding region in the ZJ0H289 genome is represented by the green and orange areas in the inner layer, respectively. The genomes of the six low‐adhesive mutants are represented by the six dotted tracks. The corresponding genes in the genome are represented by the solid lines between the tracks. For mutations occurring at the position, a synonymous mutation is represented by a gray line, a missense mutation is represented by a red line, an insertion is represented by a yellow line, and a deletion is represented by a blue line. (D) Mutations within *cpxA* include the L168 deletion (L1, L2, and L3), the V26 and L27 insertion (L5 and L6), and the R410C substitution (L4). (E) Representative images showing the spotting of surface‐bound ZJ0H289, ZJ0H289*cpxA* ^*I380V*^, ZJ0H289*cpxA* ^*L168del*^, ZJ0H289*cpxA* ^*V94M*^, ZJ0H289*cpxA* ^*V26_L27ins*^, and ZJ0H289*cpxA* ^*R410C*^. The dilution factors were 5, 50, 500, and 1000, respectively. (F) Bacterial counts of ZJ0H289, ZJ0H289*cpxA* ^*L168del*^, ZJ0H289*cpxA* ^*V26_L27ins*^, and ZJ0H289*cpxA* ^*R410C*^ enumerated from feces. The values represent mean ± SEM, *n* = 5 mice per group, two‐tailed Mann–Whitney test. **p < 0.01; *p < 0.05; ns, not significant.

Experimental evolution has been proven to be a powerful strategy for investigating the environmental adaptation of microbes^18^, ^19^. In this study, we also adopted this strategy to define bacterial genes involved in the adhesion and colonization of *K. pneumoniae*. ZJ0H289 was chosen as the starting strain, as it showed strong surface attachment and steady gut colonization in mice. ZJ0H289 was continuously cultured for 2 weeks. The resulting culture was then statically incubated in a polystyrene plate for 1 h and the planktonic bacterial cells in the supernatant were transferred to a new polystyrene plate for another round of incubation. This procedure was repeated, and the remaining planktonic bacteria were plated onto the agar plates (Figure 2A).

Six single colonies were randomly picked, validated as low‐adhesive derivatives (Figures 2B and S3), and whole‐genome sequenced. We then compared the genome of these derivatives (named ZJ0H289‐L1 to ‐L6) with their parental strain ZJ0H289 and found multiple synonymous, missense, insertion, and deletion mutations (Figure 2C). As synonymous mutations normally result in no mutagenic effect, these mutations were not further analyzed. Other three mutation types occurred in five genes, including *cpxA*, *phoA*, *sbmA*, *cydA*, and *lysN*. Derived strains ZJ0H289‐L1, ZJ0H289‐L2, and ZJ0H289‐L3 share the same mutations in *cpxA*, *sbmA*, *cydA*, and *lysN*; they possibly evolved from the same lineage. Similarly, ZJ0H289‐L5 and ZJ0H289‐L6 share the same mutations in *cpxA* and *phoA*. Notably, *cpxA* mutations were found in all six isolates and occurred at three different positions (Figure 2D), implying that *cpxA* is a strong candidate related to *K. pneumoniae* colonization.

### Mutated *cpxA* produces functional CpxA variants

Next, we validated these candidate genes by deleting a segment in each open reading frame (ORF) of *cpxA*, *phoA*, *sbmA*, *cydA*, and *lysN* in the genome of ZJ0H289. Surprisingly, all five gene knockout mutants, including ZJ0H289*∆cpxA*, ZJ0H289*∆phoA*, ZJ0H289*∆sbmA*, ZJ0H289*∆cydA*, and ZJ0H289*∆lysN*, failed to show attenuated surface attachment to the polystyrene plates (Figure S4A,B), suggesting that the altered adhesion is not due to the loss of these genes. Thus, we re‐analyzed the obtained mutations in our experimental evolution and noticed that although three different mutation types (missense, insertion, and deletion) occurred in *cpxA*, none of them indeed disrupt the ORF of *cpxA* (Figure 2D). In other words, these mutated *cpxA* genes could still produce intact and presumably functional CpxA variant proteins (gene sequences in ZJ0H289 are referred to as the references in this study).

Instead of disrupting the *cpxA* gene, we next introduced mutations into ZJ0H289 and generated three mutant strains, namely, ZJ0H289*cpxA* ^*L168del*^ (*cpxA:502_504delCTG*), ZJ0H289*cpxA* ^*V26_L27ins*^ (*cpxA:69_74insAGTGCT*), and ZJ0H289*cpxA* ^*R410C*^ (*cpxA:1228C*>*T*). All three mutants showed similar growth rates in the normal media compared to the WT strain, while the expression levels of their *cpxA* transcripts were partly increased (Figure S5A,B). As expected, these mutants behaved as phenocopies of ZJ0H289‐L1 to ‐L6, showing low surface adhesion (Figures 2E and S5C). More importantly, ZJ0H289*cpxA* ^*L168del*^, ZJ0H289*cpxA* ^*V26_L27ins*^, and ZJ0H289*cpxA* ^*R410C*^ also showed attenuated gastrointestinal colonization in mice compared to ZJ0H289 (Figure 2F). Therefore, we suggest that these CpxA variants result in reduced surface adhesion and intestinal colonization of the bacterium.

### CpxRA negatively regulates the type 3 fimbriae expression

**Figure 3:**
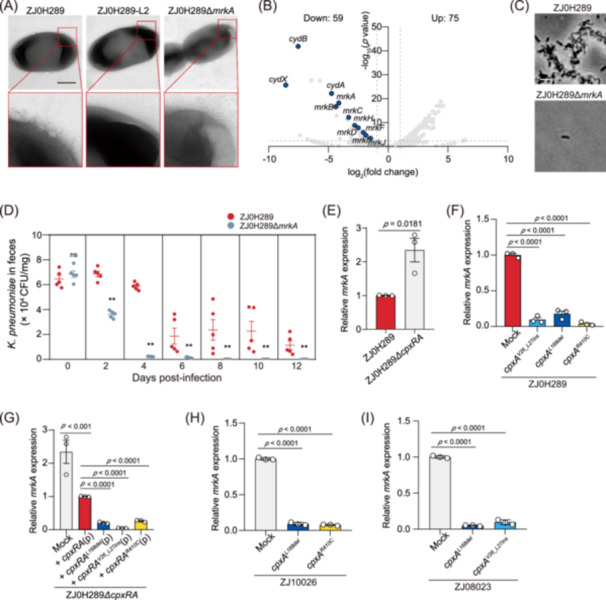
CpxA regulates type 3 fimbriae to affect *K. pneumoniae* colonization. (A) Transmission electron microscope analysis of ZJ0H289, ZJ0H289‐L2, and ZJ0H289Δ*mrkA*. Fimbriae can be observed on the surface of ZJ0H289, but not on ZJ0H289‐L2 and ZJ0H289Δ*mrkA*. The scale bar represents 500 nm. (B) Volcano plots of differentially expressed genes of the ZJ0H289‐L2 compared to the ZJ0H289. The blue dots represent genes with downregulated transcription levels. The volcano plot depicts log_2_(fold change) on the x‐axis and ‐log_10_(*P* value) on the y‐axis. (C) The representative images showing the adhesion ability of ZJ0H289 and ZJ0H289Δ*mrkA*. The scale bar represents 10 μm. (D) Bacterial counts of ZJ0H289 and ZJ0H289Δ*mrkA* enumerated from feces. The values represent mean ± SEM, *n* = 5 mice per group, two‐tailed Mann–Whitney test. **p < 0.01; *p < 0.05; ns, not significant. (E) Relative expression of *mrkA* in ZJ0H289 and ZJ0H289Δ*cpxRA*. (F) Relative expression of *mrkA* in wild‐type (WT) ZJ0H289, ZJ0H289*cpxA* ^*L168del*^, ZJ0H289*cpxA* ^*V26_L27ins*^, and ZJ0H289*cpxA* ^*R410C*^. (G) Relative expression of *mrkA* in ZJ0H289Δ*cpxRA* and ZJ0H289Δ*cpxRA* complemented with *cpxRA, cpxRA* ^*L168del*^, *cpxRA* ^*V26_L27ins*^, *cpxRA* ^*R410C*^ with plasmid (marked by (p) in the chart). (H) Relative expression of *mrkA* in ZJ10026, ZJ10026*cpxA* ^*L168del*^, and ZJ10026*cpxA* ^*R410C*^. (I) Relative expression of *mrkA* in ZJ08023, ZJ08023*cpxA* ^*L168del*^, and ZJ08023*cpxA* ^*V26_L27ins*^. For (E) to (I), values indicate mean ± SEM, *n* = 3, Student's *t*‐test.

The next question is how CpxA‐mediated regulation affects the adhesion and colonization of *K. pneumoniae*. Using transmission electron microscopy (TEM), we observed a drastic loss of type 3 fimbriae in the evolved ZJ0H289 compared to their parental strain (Figures 3A and S6). Consistently, transcriptome analysis showed downregulated transcription of the type 3 fimbria encoding genes, such as *mrkA*, *mrkB*, *mrkC*, *mrkD*, *mrkF*, *mrkH*, *mrkI*, and *mrkJ*, in ZJ0H289‐L2 compared to ZJ0H289 (Figure 3B). We postulate that the reduced type 3 fimbriae expression is the direct cause of lowered surface adhesion and gut colonization of *K. pneumoniae*. To verify this, we generated an *mrkA* gene knockout that has no type 3 fimbriae (Figure 3A) and named it ZJ0H289*∆mrkA*. As expected, ZJ0H289*∆mrkA* showed reduced surface adhesion and mouse gut colonization when compared to ZJ0H289 (Figures 3C,D and S7).

CpxA is a sensor kinase of the two‐component system CpxRA that may respond to envelope stress and control various gene expressions in *Escherichia coli* ^20^, ^21^, ^22^. A recent study reported that CpxRA negatively regulates the expression of type 3 fimbria in *K. pneumoniae* ^23^. We also showed that the deletion of the entire *cpxRA* gene locus enhanced the transcription of *mrkA* in ZJ0H289 (Figure 3E), confirming that Cpx is a negative regulator of *mrk* genes. In contrast, ZJ0H289 derivates containing specific *cpxA* mutations (*cpxA* ^*L168del*^, *cpxA* ^*V26_L27ins*^, and *cpxA* ^*R410C*^) showed lowered expression levels of the *mrkA* transcript. (Figure 3F). We further utilized plasmids containing variant *cpxRA* genes (*cpxRA*, *cpxRA* ^*L168del*^, *cpxRA* ^*V26_L27ins*^, or *cpxRA* ^*R410C*^) to complement ZJ0H289*∆cpxRA*. While the *cpxRA* complement suppressed the *mrkA* transcription, *cpxRA* ^*L168del*^, *cpxRA* ^*V26_L27ins*^, and *cpxRA* ^*R410C*^ complements showed stronger inhibition of *mrkA* with similar *cpxRA* transcription (Figures 3G and S8), suggesting that these CpxA mutants are functional. Because the expression of *mrkA* is controlled by its transcriptional activator MrkH^24^, we investigated whether CpxRA regulates the *mrkA* transcription directly or through *mrkH*. We found that *mrkH* transcription was suppressed in the above *cpxA* mutants (Figures S9A). We also disrupted the *mrkH* gene in ZJ0H289 and ZJ0H289*cpxA* ^*L168del*^ and barely detected *mrkA* transcription in both *mrkH* knockout strains (Figure S9A,B). Therefore, CpxRA likely controls the expression of *mrkA* by regulating the transcription of *mrkH*. To further prove that this regulation is general in *K. pneumoniae*, we picked two other strains (ZJ08023 and ZJ10026) and introduced the above *cpxA* mutations into their genomes. As expected, these mutations inhibited the transcription of *mrkA* (Figure 3H,I) and impaired the surface adhesion of ZJ08023 and ZJ10026 as well (Figure S10).

### CpxA^L168del^ is a natural variant in *K. pneumoniae*

**Figure 4:**
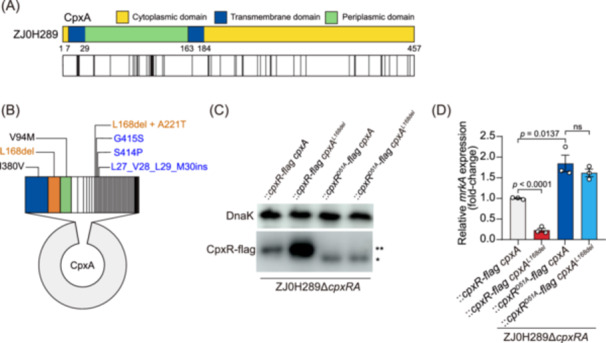
Analysis of the natural variants of *K. pneumoniae* CpxA. (A) Sequence comparison of CpxA proteins of 4072 *K. pneumoniae* strains acquired from NCBI with ZJ0H289. The vertical lines represent the positions of non‐conserved amino‐acid residues. (B) Pie chart showing the amino acid sequences of CpxA of 4072 *K. pneumoniae* strains from NCBI compared to ZJ0H289. (C) Detection of CpxR phosphorylation in ZJ0H289Δ*cpxRA::cpxR‐flag cpxA*, ZJ0H289Δ*cpxRA::cpxR‐flag cpxA* ^*L168del*^, ZJ0H289Δ*cpxRA::cpxR* ^*D51A*^ *‐flag cpxA*, and ZJ0H289Δ*cpxRA::cpxR* ^*D51A*^ *‐flag cpxA* ^*L168del*^ by immunoblot analysis. The phos‐tag acrylamide system was used to measure in vivo levels of CpxR phosphorylation. DnaK is a loading control. The single asterisk (*) indicates non‐phosphorylated CpxR, while the double asterisk (**) indicates the phosphorylated CpxR isoform. (D) Relative transcription of *mrkA* in ZJ0H289Δ*cpxRA::cpxR‐flag cpxA*, ZJ0H289Δ*cpxRA::cpxR‐flag cpxA* ^*L168del*^, ZJ0H289Δ*cpxRA::cpxR* ^*D51A*^ *‐flag cpxA*, and ZJ0H289Δ*cpxRA::cpxR* ^*D51A*^ *‐flag cpxA* ^*L168del*^. The values represent mean ± SEM, *n* = 3, Student's *t*‐test.

CpxA is an integral membrane protein consisting of a periplasmic sensor region flanked by two transmembrane domains and a C‐terminal cytosolic transmitter core region^25^ (Figure 4A). To analyze the amino acid polymorphisms of CpxA in genome‐sequenced *K. pneumoniae* strains, 4072 *K. pneumoniae* genomes were downloaded from the NCBI database and their *cpxA* genes were extracted. Using CpxA from ZJ0H289 as the reference sequence, 54 CpxA variant sequences were obtained. Interestingly, L168del seems to be common in the natural variants of CpxA, while polymorphic sequences closely related to V26_L27ins and R410C, such as L27_V28_L29_M30ins, S414P, and G415S, were also detected in the natural variants of CpxA (Figure 4B). In addition, among the 44 *K. pneumoniae* strains that we tested previously, one strain (ZJ02001) also contains a CpxA^L168del^ variant.

In the two‐component system CpxRA, CpxA phosphorylates CpxR and the activated CpxR acts as a transcriptional regulator to modulate the expression of downstream genes^20^, ^22^, ^26^, ^27^. We speculate that natural CpxA variants may result in altered *cpxRA* transcription and phosphorylated CpxR protein levels. Using L168del as an example, we showed varied levels of phosphorylated CpxR, but not CpxR^D51A^ (a non‐phosphorylated form^28^), in the presence of CpxA or CpxA^L168del^ (Figure 4C). The phosphorylated CpxR leads to upregulated *cpxR* and *cpxA* due to autoactivation^29^ (Figure S11), which explains the increase in the *cpxA* transcription of the ZJ0H289 variants. In line with altered CpxR levels, the transcription levels of *mrkA* also changed (Figure 4D).

### *K. pneumoniae* CpxA senses amino acids

Since CpxA is the sensory kinase of the two‐component system CpxRA^29^, ^30^, we next aimed to determine whether it monitors the intestinal environmental factors to regulate colonization. A recent report showed that serotonin can inhibit the Cpx signaling in enterohemorrhagic *E. coli* and *Citrobacter rodentium* ^31^. However, we showed that serotonin even slightly enhanced the Cpx signaling in *K. pneumoniae* according to the suppressed *mrkA* transcription (Figure S12).

**Figure 5:**
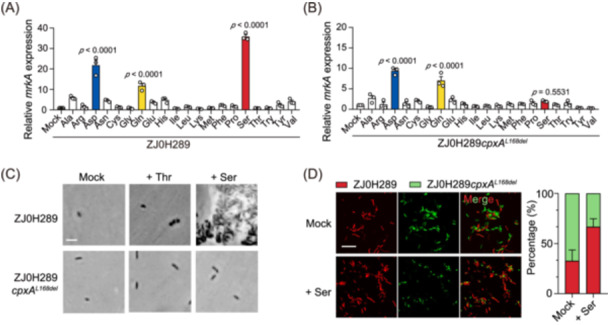
CpxA variants show varied responses to different amino acids. (A, B) Relative expression of *mrkA* in ZJ0H289 (A) or ZJ0H289*cpxA* ^*L168del*^ (B) induced with different amino acids. The values represent mean ± SEM, *n* = 3, Student's *t*‐test. (C) The representative images showing the adhesion of ZJ0H289 and ZJ0H289*cpxA* ^*L168del*^ in the presence of additional threonine (Thr) or serine (Ser). The scale bar represents 10 μm. (D) Confocal fluorescence microscopy analysis of ZJ0H289 (red) expressing mCherry and ZJ0H289*cpxA* ^*L168del*^ (green) expressing eGFP with or without the induction of serine. The panel on the right indicates the percentage of each strain. The scale bar represents 20 μm.

Therefore, we turned our attention to other small molecular metabolites in the gut. Free amino acids are highly abundant and can reach millimolar levels in the lumen of the intestines^32^. To test whether *K. pneumoniae* CpxA responds to amino acids, we measured the *mrkA* transcription in ZJ0H289 induced by different amino acids. Notably, the presence of 1 mM serine, aspartic acid, or glutamine strongly upregulated the transcription of *mrkA* (Figure 5A). We next tested ZJ0H289*cpxA* ^*L168del*^, which harbors a natural CpxA variant. Interestingly, only aspartic acid and glutamine, but not serine, enhanced the *mrkA* transcription in ZJ0H289*cpxA* ^*L168del*^ (Figure 5B). Similar results were obtained in other *K. pneumoniae* strains such as ZJ10026 and ZJ08023 (Figure S13). Consistent with the transcriptional analysis on the *mrkA* gene, ZJ0H289, but not ZJ0H289*cpxA* ^*L168del*^, showed enhanced surface adhesion in the presence of serine, but not threonine (Figure 5C).

Therefore, we postulate that the existence of natural CpxA variants, in addition to their different abilities to sense varied amino acids, may render diverse colonization ability in *K. pneumoniae* at the species level. To demonstrate this, we mixed fluorescently labeled ZJ0H289 (red) and ZJ0H289*cpxA* ^*L168del*^ (green) at a 1:10 ratio and performed a surface attachment assay. Under two different medium conditions (with and without serine), a noticeable change in the proportion of ZJ0H289 and ZJ0H289*cpxA* ^*L168del*^ in the total adherent population was observed (Figure 5D).

### Cpx‐mediated serine sensing affects gut colonization of *K. pneumoniae* in mice

**Figure 6:**
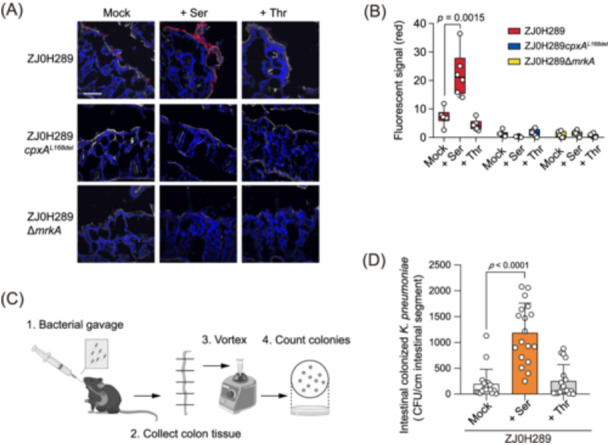
Serine induction enhances the intestinal colonization of *K. pneumoniae* in mice. (A) Representative images of ZJ0H289, ZJ0H289 *cpxA* ^*L168del*^, and ZJ0H289Δ*mrkA* colonization in the presence of serine or threonine in the ex vivo mouse intestine ligation assays. The red signal indicates the colonized *K. pneumoniae*. F‐actin was stained with phalloidin (gray) and cell nuclei were stained with Hoechst (blue). The scale bar represents 50 μm. (B) The box and whisker plots showing the calculated red fluorescent signals in (A), *n* = 6, and *p* values were calculated using Student's *t*‐test. (C) Schematic diagram of the procedure for measuring the intestinal colonized *K. pneumoniae* 8 h post‐gavage (created in BioRender. Tao, L. (2025) https://BioRender.com/o34y527). (D) The numbers of intestinal colonized ZJ0H289 induced with serine or threonine. The values represent mean ± SEM, *n* = 18 mice per group, Student's *t*‐test.

To investigate Cpx‐mediated colonization of *K. pneumoniae* on mouse intestinal epithelium, we performed the ex vivo colonization assay in ligated mouse intestines. ZJ0H289 was readily attached to the surface of the mouse intestinal epithelium. The number of bound bacteria further increased in the presence of 1 mM serine, but not threonine. In contrast, ZJ0H289*cpxA* ^*L168del*^ was only weakly bound to the intestinal epithelium, regardless of the presence of serine or threonine (ZJ0H289*ΔmrkA* served as a negative control, Figure 6A,B). Lastly, we assessed the potential effect of dietary amino acids on the intestinal colonization of *K. pneumoniae* in mice. Mice were fasted, inoculated with *K. pneumoniae* ZJ0H289 via oral gavage, and provided with drinking water supplemented with serine or threonine, but no food. The number of intestinal colonized *K. pneumoniae* was measured 8 h postinoculation (Figure 6C). More *K. pneumoniae* were detected in the intestines of mice fed with serine water, when compared to mice fed with normal water or threonine water, suggesting that high free serine may enhance the gut colonization of *K. pneumoniae* (Figure 6D).

## DISCUSSION

Over the past decades, *K. pneumoniae* has emerged as a major clinical and public health threat owing to its high prevalence in communities and hospitals^33^, ^34^. Gastrointestinal carriage is thought to be a major reservoir of *K. pneumoniae* ^3^, as the reported gut colonization rate varied from ~20% up to 77% in different clinical settings^10^, ^15^, ^35^. Here, we report that CpxA‐mediated amino acid sensing regulates the type 3 fimbriae expression and thus affects the intestinal colonization of *K. pneumoniae*. We also show that natural CpxA variants have varied signaling activities and responses to different amino acids, thus diversifying the environmental colonization potential of the bacterium.

CpxRA is a two‐component regulatory system consisting of the sensor histidine kinase CpxA and the response regulator CpxR^20^. It responds to multiple envelope stress, such as altered pH, osmotic strength, and misfolded proteins. In different bacteria, CpxRA may modulate the expression of various genes involved in inner membrane‐associated processes, including envelope protein folding and degrading, envelope maintenance, envelope‐localized protein complexes, metabolic maintenance, other regulators, and virulence factors^36^, ^37^, ^38^, ^39^. Recent studies demonstrated that CpxRA in enterohemorrhagic *E. coli* and *C. rodentium* could sense the neurotransmitter serotonin to regulate the virulence of these enteric bacteria^31^, ^40^. However, serotonin failed to suppress the signaling of *K. pneumoniae* CpxA. Instead, we found that *K. pneumoniae* CpxA responded to amino acids, such as serine, aspartic acid, and glutamine. This could be due to the differences in the sequence similarity of CpxA. CpxR/A sequences from *E. coli* 86‐24 and *C. rodentium* DBS770 exhibit high similarity, with similarities of 97.00% and 98.69% for CpxR and CpxA, respectively, while they both show ~95% similarities with CpxR/A from *K. pneumoniae* ZJ0H289 (Figure S14). More interestingly, different CpxA variants showed different selectivity on their sensing substrates: the reference CpxA sensed serine, aspartic acid, and glutamine, while CpxA^L168del^ sensed aspartic acid and glutamine but no longer serine. Notably, CpxA^L168del^ is a naturally existing variant of *K. pneumoniae*. These results suggest that Gram‐negative bacterial CpxA variants may have the potential to monitor different small‐molecule metabolites during environmental adaptation.

We next show that CpxRA negatively regulates the transcription of *mrk* genes in *K. pneumoniae*, which is in agreement with a recent study showing that the CpxRA‐regulated small RNA RyhB negatively affects the expression of type 3 fimbriae in *K. pneumoniae* CG43^23^. RyhB is negatively regulated by iron through the ferric uptake regulator (Fur), which also represses another regulator, *iroP*, that downregulates the type 3 fimbriae expression in *K. pneumoniae* ^41^. Another study reported that cyclic adenosine monophosphate (cAMP) regulates type 3 fimbriae expression through the cyclic‐di‐GMP signaling pathway^42^. On the other hand, we found that the *mrkH* operon, which encodes regulators for the *mrkA* operon^24^, ^43^, was downregulated in our CpxA mutants. It seems that CpxRA may influence *mrkA* expression in multiple ways, which remains to be further studied. Fimbriae were previously reported to be important to the virulence and biofilm formation of *K. pneumoniae* ^44^, ^45^. We further demonstrated that the type 3 fimbriae contribute to the intestinal colonization of *K. pneumoniae*.

**Figure 7:**
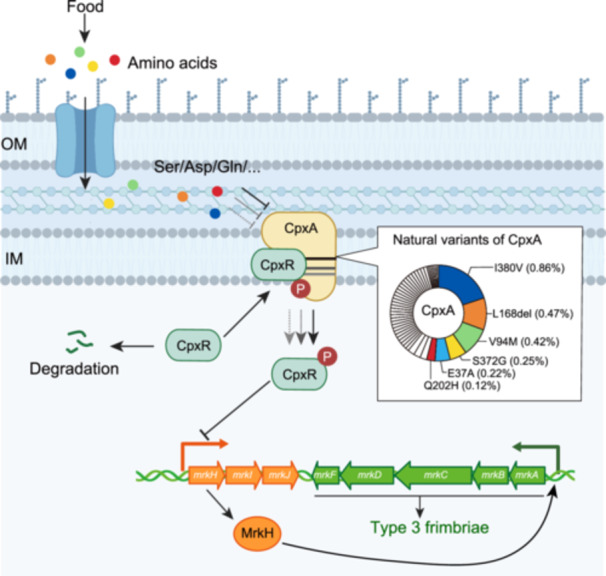
The proposed model for Cpx‐mediated regulation on *K. pneumoniae* colonization (created in BioRender. Tao, L. (2025) https://BioRender.com/u82e872). Small molecules such as amino acids enter into the periplasm of *K. pneumoniae*. CpxA responds to the amino acids to affect the autophosphorylation of CpxA. CpxA variants (as analyzed in Figure 4B) have varied activities and lead to different levels of CpxR phosphorylation. The phosphorylated CpxR acts as a repressor of *mrk* genes and thus affects the adhesion and colonization of the bacteria.

Taken together, we suggest that CpxA‐mediated signaling diversifies the surface adherence and intestinal colonization of *K. pneumoniae* at two levels (Figure 7): (1) CpxA variants have different phospho‐transfer activities in regulating CpxR and thus affect the basal transcription of *mrk* genes; (2) CpxA variants show varied responses to different amino acids (and possibly other gut metabolites). Such diversity may confer potential adaptability to *K. pneumoniae* at the population level, allowing its different individuals to better colonize in varied environments. Since amino acids are major nutrients as well as abundant food digestion products in the gut, amino acid sensing could be a strategy for *K. pneumoniae* to monitor the nutritious intestinal environment and initiate colonization. Finally, we suggest that CpxA could serve as a potential drug target for the development of therapeutics to eradicate intestinal colonized *K. pneumoniae* in both patients and asymptomatic carriers.

## MATERIALS AND METHODS

### Bacterial strains and growth conditions

The bacterial strains used in this study are listed in Table S1. *K. pneumoniae* and *E. coli* were routinely cultured in LB broth at 37°C unless otherwise specified. To prepare the solid medium, 1.5% (w/v) agar was used. The following antibiotics were added to the medium as necessary: 100 µg/ml hygromycin B (for *K. pneumoniae* and *E. coli*) or 50 µg/ml apramycin (for *K. pneumoniae*).

### Mice

The C57BL/6 mice (male, 6–8 weeks old) used in this study were purchased from the Laboratory Animal Resources Center of Westlake University (Hangzhou, China). Mice were routinely maintained by full‐time staff in specific‐pathogen‐free micro‐isolator cages at 20–24°C with 40%–60% humidity. They were exposed to 12:12 light–dark cycles and given free access to food and water.

### MLST analysis of *K. pneumoniae*

The neighbor‐joining evolutionary tree was constructed based on the core genome MLST of clinical *K. pneumoniae* strains^46^, and SeqSphere+ software was used to draw the tree.

### Bacterial surface attachment assay

Overnight‐grown *K. pneumoniae* culture was replaced with M9 medium and transferred to a polystyrene 24‐well culture plate (Corning Costar) and incubated at 37°C for 90 min. The culture was then removed and the plate was washed three times with phosphate‐buffered saline (PBS). The surface‐bound bacteria were either observed under a microscope (Olympus IX73) or scraped down, serially diluted, and spotted on LB agar plates. For fluorescent labeling of bacteria, ZJ0H289 and ZJ0H289 *cpxA* ^*L168del*^ were transformed with plasmids containing genes encoding either mCherry or eGFP.

### Intestinal colonization of *K. pneumoniae* in mice

Mice were consecutively given drinking water containing an antibiotic cocktail (0.045 g/l vancomycin, 0.4 g/l kanamycin, and 0.215 g/l metronidazole) for 6 days and switched to regular drinking water 1 day before infection. Each mouse was administered ~10^8^ CFU *K. pneumoniae* (200 μl of bacterial culture at OD_600_ = 1.0) by oral gavage. Feces from the mice were collected at the indicated time points. For assessment of *K. pneumoniae* in the feces, 1 μg of feces was resuspended in 1 ml of PBS. 50 μl of the resuspension was then spread onto a selective agar plate for colony counting.

### Experimental evolution of *K. pneumoniae*

*K. pneumoniae* strain ZJ0H289 was cultured in LB medium with daily passages for 2 weeks. After that, 2 ml of bacterial culture was transferred to a six‐well polystyrene plate, replaced with M9 medium, and incubated at 37°C for 1 h. 500 μl of the supernatant was taken for another round of culturing overnight. The process of in vitro binding of the bacteria was repeated five times, the planktonic cells in the supernatant were spread onto an LB agar plate, and colonies were picked the next day for further studies.

### TEM

Ultrapure water droplets containing *K. pneumoniae* cells were transferred to a copper mesh. The mesh was repeatedly dipped in ultrapure water for 2 s each time and stained with 0.05% uranyl acetate for 15 s. The mesh was then air‐dried and scanned by a 120 kV transmission electron microscope (CRYO‐EM005; Thermo Fisher Scientific).

### Genetic operation of *K. pneumoniae*

Construction of the insertional mutant was performed using lambda‐red‐based technology as previously described^47^. Briefly, an apramycin‐resistant gene flanked by the homologous regions of the gene of interest was generated with PCR. *K. pneumoniae* cells were transformed with plasmid pACBSR‐hyg, induced with l‐arabinose for lambda‐red recombinase expression, followed by the electroporation of the above PCR product. The bacteria were selected with hygromycin and apramycin on an LB agar plate. The isogenic and single‐base mutants were generated using the CRISPR/Cas9‐assisted lambda‐red system as previously described^48^. Briefly, *K. pneumoniae* cells were transformed with plasmid pCasKP and induced with L‐arabinose. A plasmid containing the guide RNA and the repair template of the gene of interest was electroporated into the above *K. pneumoniae* cells. Bacterial colonies were selected on an LB agar containing hygromycin and apramycin. The success of gene knockout was verified by PCR and DNA sequencing.

### Colonization of amino acid‐induced *K. pneumoniae*

Experimental mice were fasted for 12 h. Bacterial culture was induced with 1 mM amino acid for 1 h. 200 μl of the bacterial culture at OD_600_ = 1.0 (~10^8^ CFU) was administered to each fasted mouse by oral gavage. Experimental mice were provided with drinking water supplemented with corresponding amino acids, but no food. After 8 h, mice were killed, and their intestines were excised, cut into small fragments, and washed with PBS three times (5 min each). Each intestinal tissue segment was added to a centrifuge tube containing 1 ml of PBS and vortexed. 50 μl of the supernatant was spread onto a selective agar plate for colony counting.

### RNA extraction and RT‐qPCR

RNA was extracted using the Bacteria RNA Extraction Kit according to the manufacturer's protocols (Vazyme). For reverse transcription, 1 μg of extracted RNA was converted into cDNA. The real‐time PCR was conducted with Jena Qtower3G (Jena). The data were collected using qPCRsoft v4.0.8.0, normalized to endogenous 16S rRNA levels, and analyzed using the delta‐delta Ct method.

### Sequence analysis

Fully assembled and annotated genome sequences of *K. pneumoniae* strains (4072 in total) were downloaded from the NCBI. The CpxA sequences in the protein files of each strain were extracted to remove repeated sequences, and the remaining unique sequences were compared with the CpxA sequences of the wild‐type ZJ0H289 strain using CLC Sequence Viewer.

### Detection of phosphorylated CpxR

To differentiate phosphorylated and nonphosphorylated CpxR, proteins of bacterial whole lysate were separated by manganese(II) Phos‐Tag™ SDS‐PAGE (FUJIFILM Wako Pure Chemical Corporation). Then, the Phos‐Tag protein gel was incubated in Towbin buffer containing 1 mM ethylenediaminetetraacetic acid for 10 min at room temperature with gentle shaking, followed by washing in Towbin buffer for 15 min. The treated Phos‐Tag protein gel was then applied to immunoblot analysis.

### Immunoblot analysis

100 μl of overnight bacterial culture was harvested and resuspended with 100 μl of SDS sample buffer and boiled for 5 min. 10 μl of protein sample was separated by SDS‐PAGE and transferred to a nitrocellulose membrane (GE Healthcare, #10600002). Western blot assays were performed using a standard procedure with the following primary antibodies: mouse monoclonal anti‐FLAG antibody (#HA601167, HUABIO) and rabbit polyclonal anti‐DnaK antibody (CSB‐PA633459HA01EGW, CUSABIO). The blots were developed using the enhanced chemiluminescence substrate (Thermo Fisher Scientific, #1863096); the signals were detected using a gel imaging system (AI680RGB, GE Healthcare).

### Bacterial colonization in ligated intestines

The experimental procedure was modified from a previous intestine‐loop ligation assay^49^. In brief, mice were euthanized, and their intestines were excised, sealed into ~2 cm segments with silk ligatures, and injected with ~10^8^ CFU of *K. pneumoniae*. The intestinal segments were cultured in an incubator at 37°C for 90 min. Both ends of the mouse intestinal segment were cut, washed 3 times with PBS, fixed with 4% paraformaldehyde for 15 min, and then washed with PBS. The intestinal tissue was embedded with the OCT cryosection embedding agent for sectioning.

### Tissue section and fluorescent staining

The tissue samples embedded in OCT were cut into 10 μm thick sections and stained with Hoechst and phalloidin. Fluorescent images were captured using a scanning confocal microscope (Olympus FV3000) with the software FV31S‐SW v2.3.2.169.

## AUTHOR CONTRIBUTIONS

**Danyang Li**: Conceptualization (equal); data curation (equal); formal analysis (lead); investigation (lead); methodology (equal); validation (lead); visualization (equal); writing—original draft (equal); writing—review and editing (equal). **Qiucheng Shi**: Data curation (equal); formal analysis (equal); investigation (equal); methodology (equal); resources (equal); software (equal). **Liuqing He**: Data curation (equal); formal analysis (equal); investigation (equal); software (equal). **Jianhua Luo**: Formal analysis (equal); validation (equal); visualization (equal). **Huajie Zhu**: Investigation (equal). **Xiaoting Hua**: Data curation (equal); resources (equal). **Yunsong Yu**: Resources (equal); supervision (equal). **Yan Jiang**: Data curation (equal); methodology (equal); resources (equal); software (equal); supervision (equal); writing—review and editing (equal). **Liang Tao**: Conceptualization (lead); data curation (equal); formal analysis (equal); funding acquisition (lead); methodology (equal); project administration (lead); resources (equal); supervision (lead); visualization (equal); writing—original draft (equal); writing—review and editing (lead).

## ETHICS STATEMENT

All animal procedures in this study were approved by the Institutional Animal Care and Use Committee (IACUC) at Westlake University under Protocol #22‐040‐TL‐3. Mice administered bacterial gavage were monitored every 12 h. Animals were immediately killed if they developed any of the following signs: difficulty breathing, disorientation, or inability to move upon gentle stimulation.

## CONFLICT OF INTERESTS

The authors declare no conflict of interests.

## Supporting information

Supporting information.

## Data Availability

The genomes of *K. pneumoniae* strains used in this study have been deposited to the NCBI database (BioProject PRJNA1021041, PRJNA1145135, and PRJNA1145120). Other data that support the findings of this study are available from the corresponding author upon request. Source data are provided in the article.
