# Supplementary material for: Cpx‐mediated amino acid sensing diversifies gastrointestinal colonization of Klebsiella pneumoniae
Source: mLife. 2025 Apr 23;4(2):181–92. doi: 10.1002/mlf2.70005 (PMC12042121; doi:10.1002/mlf2.70005)
Supplement: Supplementary file 1 — Supporting information. [file MLF2-4-181-s001.pdf]

Supplementary information

# Cpx-mediated amino acid sensing diversifies gastrointestinal colonization of *Klebsiella pneumoniae*

Danyang Li[1,2,3,4], Qiucheng Shi[5], Liuqing He[2,3,4], Jianhua Luo[2,3,4], Huajie Zhu[2,3,4], Xiaoting Hua[5], Yunsong Yu[5], Yan Jiang[5,*], Liang Tao[1,2,3,4,*]

[1]College of Life Sciences, Zhejiang University, Hangzhou, 310058, China

[2]Key Laboratory of Multi-omics in Infection and Immunity of Zhejiang Province and Center for Infectious Disease Research, School of Medicine, Westlake University, Hangzhou, 310024, China

[3]School of Life Sciences, Westlake University, Hangzhou, 310030, China

[4]Westlake Laboratory of Life Sciences and Biomedicine, Hangzhou, 310024, China

[5]Department of Infectious Diseases, Sir Run Run Shaw Hospital, Zhejiang University School of Medicine, Hangzhou, 310016, China

Corresponding to:

Yan Jiang, Ph. D. Email: jiangy@zju.edu.cn

Liang Tao, Ph.D. Email: taoliang@westlake.edu.cn

# Supplementary Figures

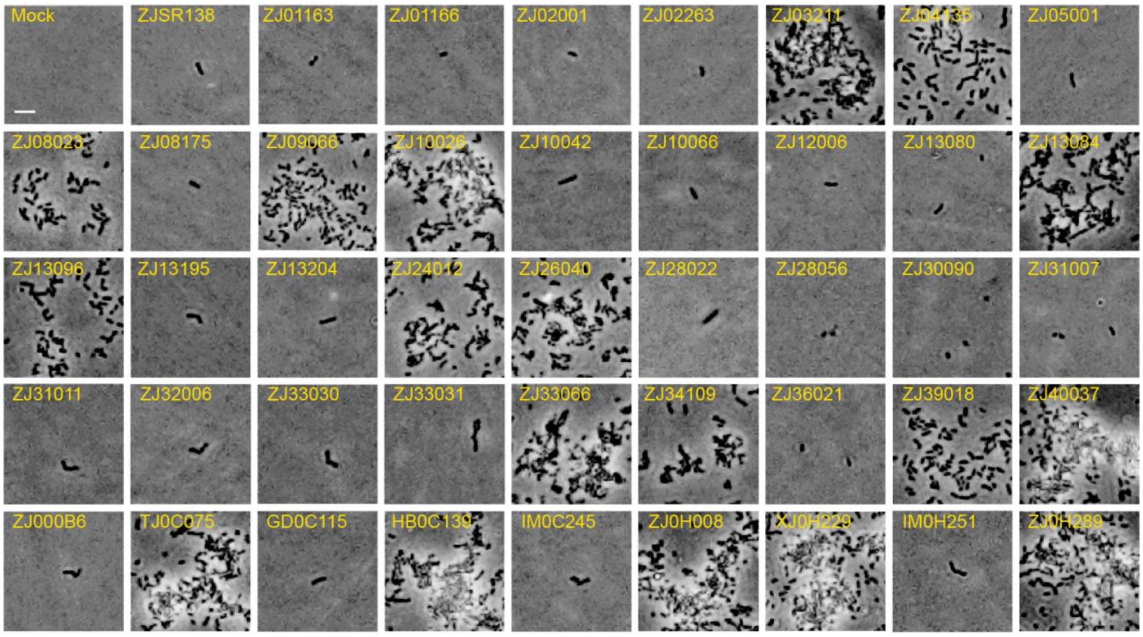


**Supplementary Fig. 1 |** Some clinical *K. pneumoniae* strains show strong surface binding ability. The representative microscopy images show the surface binding of forty-four clinical *K. pneumoniae* strains on culture plates. The scale bar represents 10 μm.

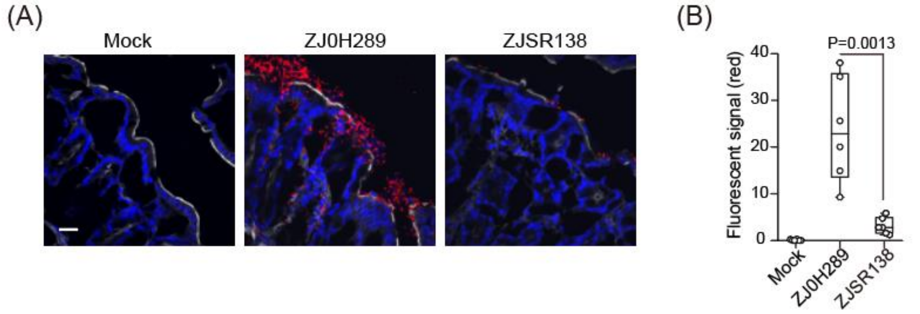


**Supplementary Fig. 2** | The *ex vivo* experiment shows that ZJ0H289 robustly binds to mouse intestinal epithelium. (A) Immunofluorescent images show that ZJ0H289 and ZJSR138 (red signals) have varied *ex vivo* binding to the mouse colonic epithelium. F-actin was stained with phalloidin (gray) and cell nuclei were stained with Hoechst (blue). The scale bar represents 20 μm. (B) The box and whisker plots show the calculated red fluorescent signals in (A), n=6, Student's *t*-test.

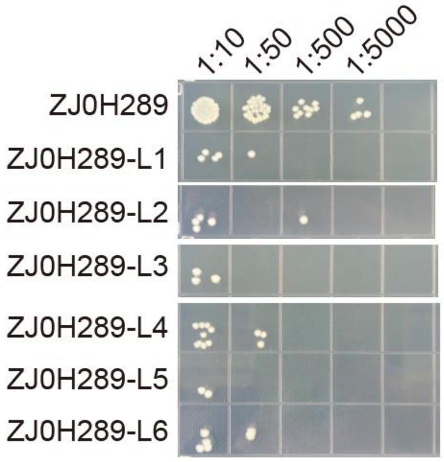


**Supplementary Fig. 3 |** ZJ0H289-L1 to -L6 have impaired surface binding ability. The representative images show the spotting of surface-bound ZJ0H289 and its mutants. The dilution factors were 10, 50, 500, and 5000, respectively.

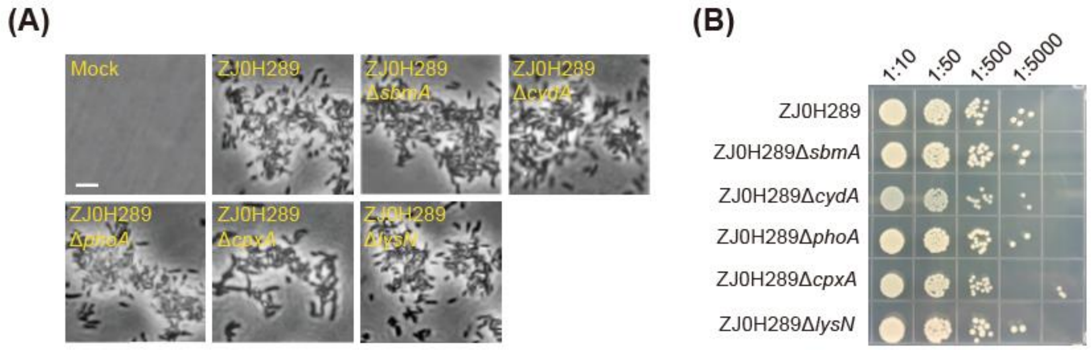


**Supplementary Fig. 4 |** Disrupting *cpxA*, *phoA*, *sbmA*, *cydA*, or *lysN* in ZJ0H289 does not reduce its surface binding ability. (A) Representative microscopy images showing the surface binding of ZJ0H289, ZJ0H289Δ*sbmA,* ZJ0H289Δ*cydA,* ZJ0H289Δ*phoA,* ZJ0H289Δ*cpxA*, and ZJ0H289Δ*lysN* on culture plates. Scale bar, 10 μm. (B) Spotting of surface bound ZJ0H289, ZJ0H289Δ*sbmA,* ZJ0H289Δ*cydA,* ZJ0H289Δ*phoA,* ZJ0H289Δ*cpxA*, and ZJ0H289Δ*lysN*. The dilution factors were 10, 50, 500, and 5000, respectively.

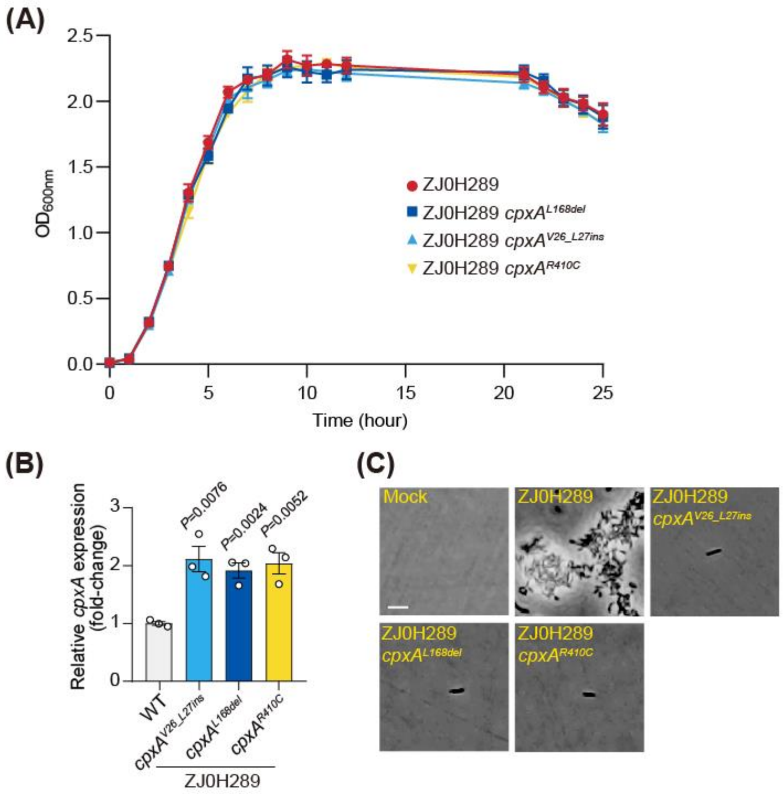


**Supplementary Fig. 5 |** Characterization of the growth and the surface adhesion of the *cpxA* mutants. (A) The growth curve of wild-type ZJ0H289, ZJ0H289 $cpxA^{L168del}$, ZJ0H289 $cpxA^{V26\_L27ins}$, and ZJ0H289 $cpxA^{R410C}$. (B) Relative expression of *cpxA* in wild-type ZJ0H289, ZJ0H289 $cpxA^{L168del}$, ZJ0H289 $cpxA^{V26\_L27ins}$ and ZJ0H289 $cpxA^{R410C}$. (C) The microscopy images of surface-bound ZJ0H289, ZJ0H289 $cpxA^{L168del}$, ZJ0H289 $cpxA^{V26\_L27ins}$, and ZJ0H289 $cpxA^{R410C}$. The scale bar represents 10 µm. The values represent mean ± SEM, n=3, Student's *t*-test.

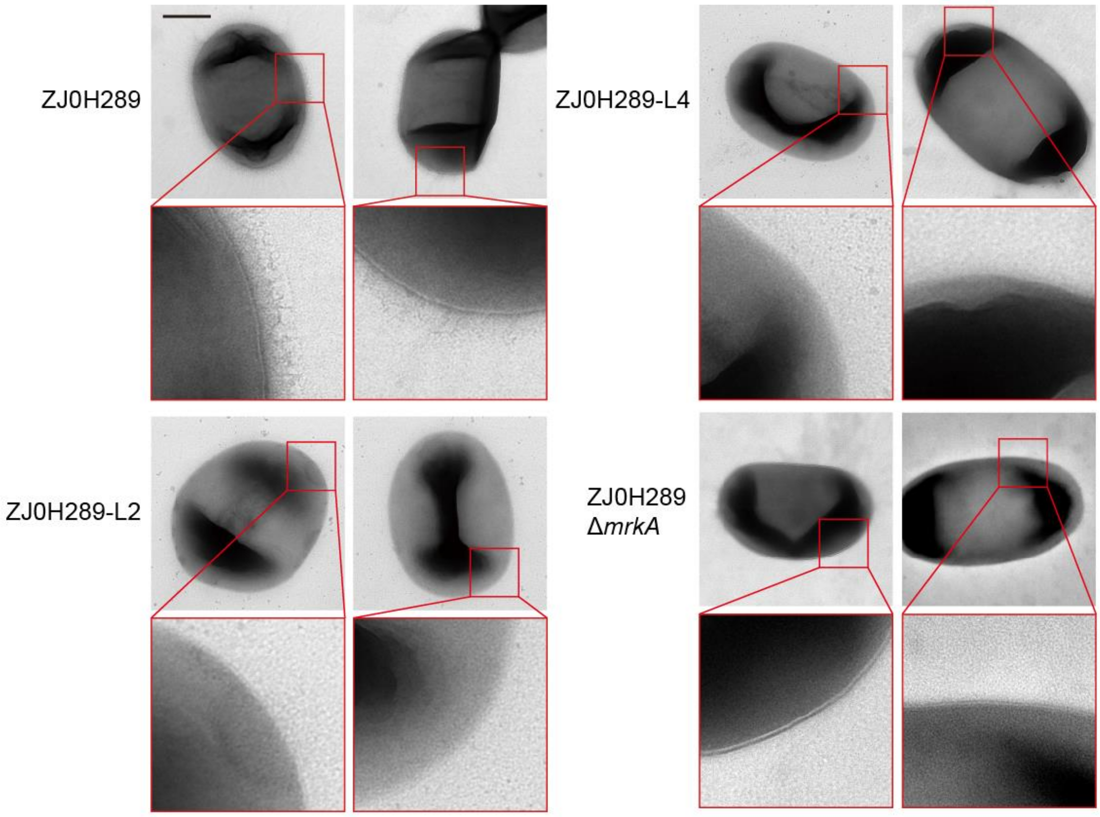


**Supplementary Fig. 6 |** Transmission electron microscope images of the ZJ0H289, ZJ0H289-L2, ZJ0H289-L4, and ZJ0H289Δ*mrkA*. Scale bar, 500 nm.

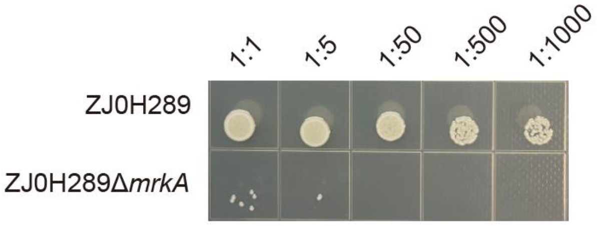


**Supplementary Fig. 7 |** Type 3 fimbriae is critical for the surface binding ability of ZJ0H289. The representative images show the spotting of surface adhesive ZJ0H289 and ZJ0H289Δ*mrkA*. The dilution factors were 5, 50, 500, and 1000, respectively.

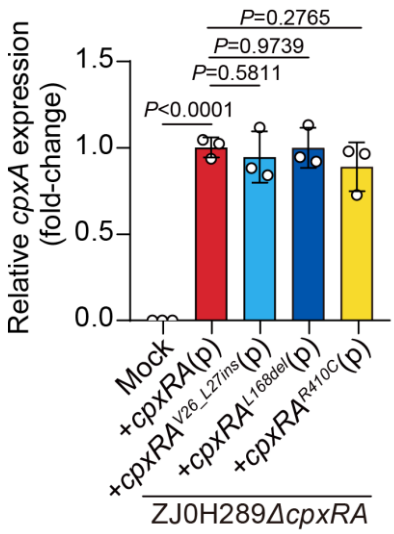


**Supplementary Fig. 8** | Validation of *cpxA* expression in the complementary strains. Relative expression of *cpxA* in ZJ0H289Δ*cpxRA* and ZJ0H289Δ*cpxRA* complemented with *cpxRA, cpxRA*$^{L168del}$, *cpxRA*$^{V26\_L27ins}$, and *cpxRA*$^{R410C}$ with plasmid, was plotted on a bar chart. The values represent mean ± SEM, n=3, Student's *t*-test.

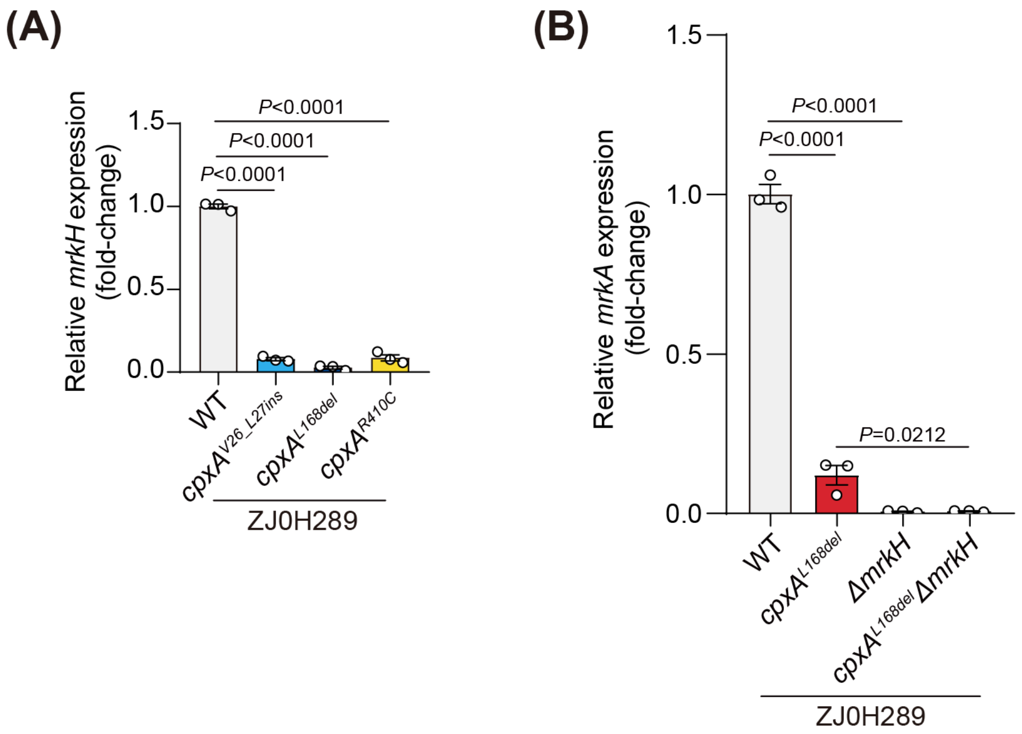


**Supplementary Fig. 9 |** Cpx signaling regulates the *mrkA* expression via *mrkH*. (A) The Relative expression of *mrkH* in wild-type ZJ0H289, ZJ0H289 *cpxA*$^{L168del}$, ZJ0H289 *cpxA*$^{V26\_L27ins}$, and ZJ0H289 *cpxA*$^{R410C}$. (B) The Relative expression of *mrkA* in wild-type ZJ0H289, ZJ0H289 *cpxA*$^{L168del}$, ZJ0H289Δ*mrkH*, and ZJ0H289 *cpxA*$^{L168del}$ Δ *mrkH*. The values represent mean ± SEM, n=3, Student's *t*-test.

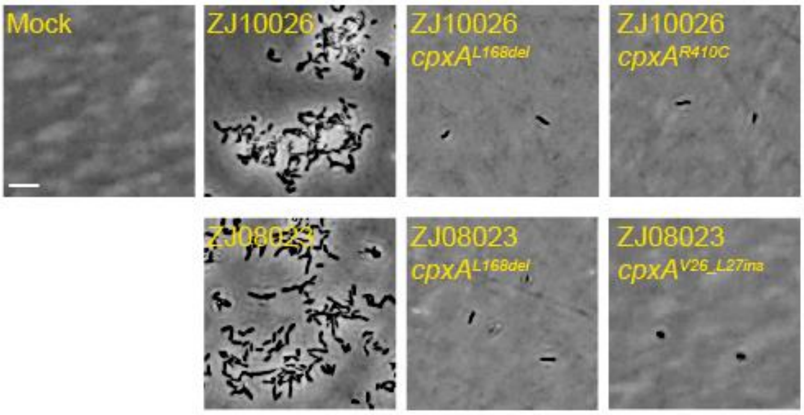


**Supplementary Fig. 10 | ** *CpxA* mutations also affect the surface adhesion of other *K. pneumoniae* strains. The microscopy images of surface-bound ZJ10026, ZJ10026 $cpxA^{L168del}$, ZJ10026 $cpxA^{R410C}$, ZJ08023, ZJ08023 $cpxA^{L168del}$, and ZJ08023 $cpxA^{V26\_L27ins}$. The scale bar represents 10 μm.

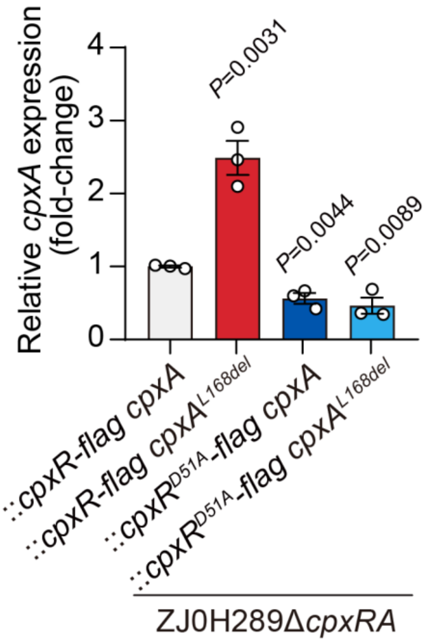


**Supplementary Fig. 11** | The phosphorylated CpxR leads to upregulated *cpxR* and *cpxA*. Relative expression of *cpxA* in ZJ0H289Δ*cpxRA::cpxR-flag cpxA*, ZJ0H289Δ*cpxRA::cpxR-flag cpxA*$^{L168del}$, ZJ0H289Δ*cpxRA::cpxR*$^{D51A}$*-flag cpxA*, and ZJ0H289Δ*cpxRA::cpxR*$^{D51A}$*-flag cpxA*$^{L168del}$. The values represent mean ± SEM, n=3, Student's *t*-test.

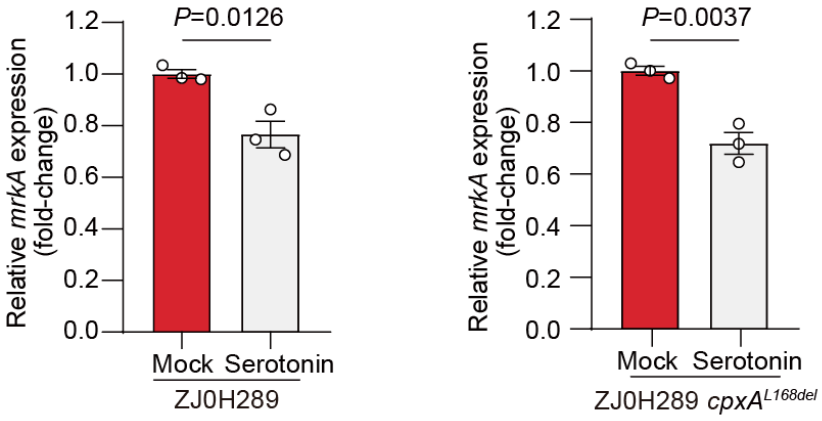


**Supplementary Fig. 12 |** Serotonin induction slightly reduces *mrkA* expression. Relative expression of *mrkA* in ZJ0H289 (left) and ZJ0H289*cpxA*$^{L168del}$ (right) with or without serotonin induction was measured and plotted on the bar charts. The values represent mean ± SEM, n=3, Student's *t*-test.

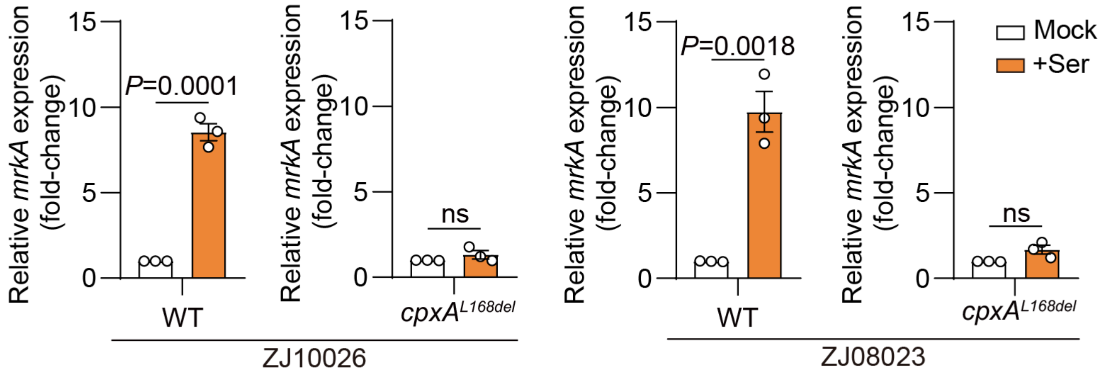


**Supplementary Fig. 13 | **Serine induction upregulates *mrkA* expression in *K. pneumoniae* strains with a reference *cpxA*, but not *cpxA*$^{L168del}$. Relative expression of *mrkA* in ZJ10026, ZJ10026*cpxA*$^{L168del}$, ZJ08023, and ZJ08023*cpxA*$^{L168del}$ with or without serine induction was measured and plotted on bar charts. The values represent mean ± SEM, n=3, Student's *t*-test.

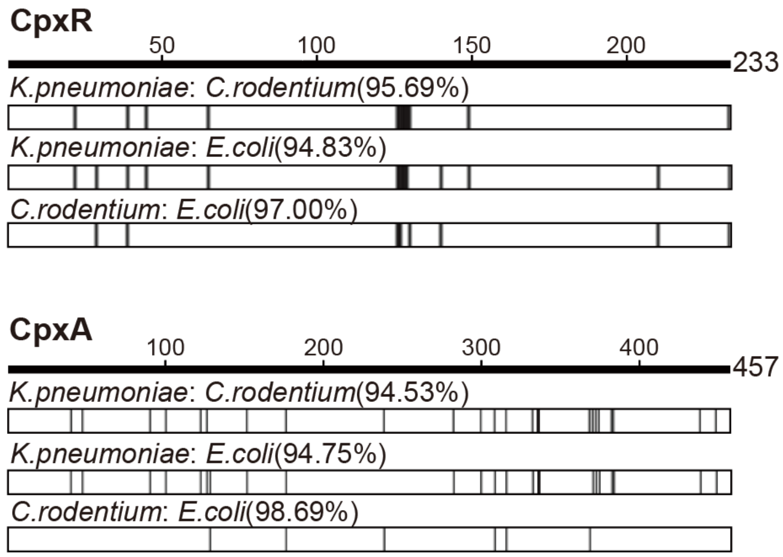


**Supplementary Fig. 14 |** Pairwise sequence comparison of CpxR and CpxA of enterohemorrhagic *E. coli* 86-24, *Citrobacter rodentium* DBS770, and *K. pneumoniae* strain ZJ0H289.

## Supplementary Table

Supplementary Table 1 Bacterial strains used in this study

| Bacterial strains | Description | Source |
|---|---|---|
| Top10 | Competent *E. coli* cell | Cwbio |
| ZJ01163 | Clinical *K. pneumoniae* strain | Zhejiang Sir Run Run Shaw Hospital |
| ZJ01166 | Clinical *K. pneumoniae* strain | Zhejiang Sir Run Run Shaw Hospital |
| ZJ02001 | Clinical *K. pneumoniae* strain | Zhejiang Sir Run Run Shaw Hospital |
| ZJ02263 | Clinical *K. pneumoniae* strain | Zhejiang Sir Run Run Shaw Hospital |
| ZJ03211 | Clinical *K. pneumoniae* strain | Zhejiang Sir Run Run Shaw Hospital |
| ZJ04135 | Clinical *K. pneumoniae* strain | Zhejiang Sir Run Run Shaw Hospital |
| ZJ05001 | Clinical *K. pneumoniae* strain | Zhejiang Sir Run Run Shaw Hospital |
| ZJ08023 | Clinical *K. pneumoniae* strain | Zhejiang Sir Run Run Shaw Hospital |
| ZJ08175 | Clinical *K. pneumoniae* strain | Zhejiang Sir Run Run Shaw Hospital |
| ZJ09066 | Clinical *K. pneumoniae* strain | Zhejiang Sir Run Run Shaw Hospital |
| ZJ10026 | Clinical *K. pneumoniae* strain | Zhejiang Sir Run Run Shaw Hospital |
| ZJ10042 | Clinical *K. pneumoniae* strain | Zhejiang Sir Run Run Shaw Hospital |
| ZJ10066 | Clinical *K. pneumoniae* strain | Zhejiang Sir Run Run Shaw Hospital |
| ZJ12006 | Clinical *K. pneumoniae* strain | Zhejiang Sir Run Run Shaw Hospital |
| ZJ13080 | Clinical *K. pneumoniae* strain | Zhejiang Sir Run Run Shaw Hospital |
| ZJ13084 | Clinical *K. pneumoniae* strain | Zhejiang Sir Run Run Shaw Hospital |
| ZJ13096 | Clinical *K. pneumoniae* strain | Zhejiang Sir Run Run Shaw Hospital |
| ZJ13195 | Clinical *K. pneumoniae* strain | Zhejiang Sir Run Run Shaw Hospital |
| ZJ13204 | Clinical *K. pneumoniae* strain | Zhejiang Sir Run Run Shaw Hospital |
| ZJ24012 | Clinical *K. pneumoniae* strain | Zhejiang Sir Run Run |

| | | |
|---|---|---|
| | | Shaw Hospital |
| ZJ26040 | Clinical *K. pneumoniae* strain | Zhejiang Sir Run Run Shaw Hospital |
| ZJ28022 | Clinical *K. pneumoniae* strain | Zhejiang Sir Run Run Shaw Hospital |
| ZJ28056 | Clinical *K. pneumoniae* strain | Zhejiang Sir Run Run Shaw Hospital |
| ZJ30090 | Clinical *K. pneumoniae* strain | Zhejiang Sir Run Run Shaw Hospital |
| ZJ31007 | Clinical *K. pneumoniae* strain | Zhejiang Sir Run Run Shaw Hospital |
| ZJ31011 | Clinical *K. pneumoniae* strain | Zhejiang Sir Run Run Shaw Hospital |
| ZJ32006 | Clinical *K. pneumoniae* strain | Zhejiang Sir Run Run Shaw Hospital |
| ZJ33030 | Clinical *K. pneumoniae* strain | Zhejiang Sir Run Run Shaw Hospital |
| ZJ33031 | Clinical *K. pneumoniae* strain | Zhejiang Sir Run Run Shaw Hospital |
| ZJ33066 | Clinical *K. pneumoniae* strain | Zhejiang Sir Run Run Shaw Hospital |
| ZJ34109 | Clinical *K. pneumoniae* strain | Zhejiang Sir Run Run Shaw Hospital |
| ZJ36021 | Clinical *K. pneumoniae* strain | Zhejiang Sir Run Run Shaw Hospital |
| ZJ39018 | Clinical *K. pneumoniae* strain | Zhejiang Sir Run Run Shaw Hospital |
| ZJ40037 | Clinical *K. pneumoniae* strain | Zhejiang Sir Run Run Shaw Hospital |
| ZJ0H289 | Clinical *K. pneumoniae* strain | Zhejiang Sir Run Run Shaw Hospital |
| ZJ0H008 | Clinical *K. pneumoniae* strain | Zhejiang Sir Run Run Shaw Hospital |
| ZJ0H229 | Clinical *K. pneumoniae* strain | Zhejiang Sir Run Run Shaw Hospital |
| ZJ0H251 | Clinical *K. pneumoniae* strain | Zhejiang Sir Run Run Shaw Hospital |
| ZJSR138 | Clinical *K. pneumoniae* strain | Zhejiang Sir Run Run Shaw Hospital |
| ZJ000B6 | Clinical *K. pneumoniae* strain | Zhejiang Sir Run Run Shaw Hospital |
| IM0C245 | Clinical *K. pneumoniae* strain | Zhejiang Sir Run Run Shaw Hospital |
| GD0C115 | Clinical *K. pneumoniae* strain | Zhejiang Sir Run Run |

| | | |
|---|---|---|
| | | Shaw Hospital |
| TJ0C075 | Clinical *K. pneumoniae* strain | Zhejiang Sir Run Run Shaw Hospital |
| HB0C139 | Clinical *K. pneumoniae* strain | Zhejiang Sir Run Run Shaw Hospital |
| ZJ0H289Δ*mrkA* | ZJ0H289 derivative, deletion of *mrkA* | This study |
| ZJ0H289Δ*sbmA* | ZJ0H289 derivative, deletion of *sbmA* | This study |
| ZJ0H289Δ*cydA* | ZJ0H289 derivative, deletion of *cydA* | This study |
| ZJ0H289Δ*cpxA* | ZJ0H289 derivative, deletion of *cpxA* | This study |
| ZJ0H289Δ*lysN* | ZJ0H289 derivative, deletion of *lysN* | This study |
| ZJ0H289Δ*phoA* | ZJ0H289 derivative, deletion of *phoA* | This study |
| ZJ0H289Δ*cpxRA* | ZJ0H289 derivative, deletion of *cpxR* and *cpxA* | This study |
| ZJ0H289-L1 | ZJ0H289 derivative, low adhesive mutants acquired from experimental evolution | This study |
| ZJ0H289-L2 | ZJ0H289 derivative, low adhesive mutants acquired from experimental evolution | This study |
| ZJ0H289-L3 | ZJ0H289 derivative, low adhesive mutants acquired from experimental evolution | This study |
| ZJ0H289-L4 | ZJ0H289 derivative, low adhesive mutants acquired from experimental evolution | This study |
| ZJ0H289-L5 | ZJ0H289 derivative, low adhesive mutants acquired from experimental evolution | This study |
| ZJ0H289-L6 | ZJ0H289 derivative, low adhesive mutants acquired from experimental evolution | This study |
| ZJ0H289$cpxA^{V26_L27ins}$ | ZJ0H289 derivative, with *69_74insAGTGCT* in the coding sequence of *cpxA* | This study |
| ZJ0H289$cpxA^{R410C}$ | ZJ0H289 derivative, with *1228C>T* in the coding sequence of *cpxA* | This study |
| ZJ0H289$cpxA^{L168del}$ | ZJ0H289 derivative, with *502_504delCTG* in the coding sequence of *cpxA* | This study |
| ZJ10026$cpxA^{L168del}$ | ZJ10026 derivative, with *502_504delCTG* in the coding sequence of *cpxA* | This study |
| ZJ10026$cpxA^{R410C}$ | ZJ10026 derivative, with *1228C>T* in the coding sequence of *cpxA* | This study |
| ZJ08023$cpxA^{L168del}$ | ZJ08023 derivative, with *502_504delCTG* in the coding sequence of *cpxA* | This study |
| ZJ08023$cpxA^{V26_L27ins}$ | ZJ08023 derivative, with *69_74insAGTGCT* in the coding sequence of *cpxA* | This study |
| ZJ0H289Δ*cpxRA::cpxR-flag cpxA* | ZJ0H289 derivative, with the addition of a 3×flag tag at the C-terminal end of CpxR | This study |
| ZJ0H289Δ*cpxRA::cpxR-flag* $cpxA^{L168del}$ | ZJ0H289 $cpxA^{L168del}$ derivative, with the addition of a 3×flag tag at the C-terminal end of CpxR | This study |
| ZJ0H289Δ*cpxRA::cpxR*$^{D51A}$-*flag cpxA* | ZJ0H289 derivative, with a mutation of the phosphorylation site of CpxR and the addition of a | This study |

| | | |
|---|---|---|
| | 3×flag tag at the C-terminal end of CpxR | |
| ZJ0H289Δ*cpxRA::cpxR*$^{D51A}$*-flag cpxA*$^{L168del}$ | ZJ0H289 *cpxA*$^{L168del}$ derivative, with a mutation of the phosphorylation site of CpxR and the addition of a 3×flag tag at the C-terminal end of CpxR | This study |
